# Editorial: Role of Diet, Physical Activity and Immune System in Parkinson's Disease

**DOI:** 10.3389/fneur.2020.611349

**Published:** 2020-12-11

**Authors:** Giovanni Albani, Salvatore Albani, Ali Keshavarzian

**Affiliations:** ^1^Department of Neurology and Neurorehabilitation, Clinical and Research Institute Auxologico Italiano, Milan, Italy; ^2^Translational Immunology, Institute, Duke-National University of Singapore Medical School, Singapore, Singapore; ^3^Division of Digestive Diseases and Nutrition, Department of Internal Medicine, Rush University Medical Center, Chicago, IL, United States

**Keywords:** diet, physical activity, immune system, Parkinson's disease, microbiome

It is believed that Parkinson's disease (PD) may originate outside of the central nervous system and converging evidence strongly suggests that gastrointestinal tract (GI) tract may be a critical system in PD pathogenesis. **(a)** The GI tract is the largest surface area and point of entry for environmental factors to interact with the host. **(b)** The microbiota that inhabit the GI tract are profoundly impacted by environmental factors including those that are known to be risk factors for PD pathogenesis such as high fat, high sugar/low fiber Western diet, and lack of physical activity [Jackson et al., (1)]. **(c)** GI tract dysfunction such as constipation is commonly observed in PD patients and often occurs decades before PD diagnosis and an abnormal microbiota (i.e., dysbiosis) is observed with those with constipation (2). **(d)** A pathological hallmark of PD, α-synuclein misfolding and aggregation, is thought to be a consequence of inflammation and the source of that inflammation could be the microbiota. Indeed, the intestinal microbiota (especially a pro-inflammatory, dysbiotic microbiota) can activate intestinal mucosal, systemic, and brain immune systems, which can culminate in neuroinflammation (3). **(e)** PD patients have microbiota dysbiosis with low levels of anti-inflammatory short chain fatty acids (SCFA) and high levels of pro-inflammatory lipopolysaccharide (LPS) (4, 5). **(f)** The GI tract is continuously inundated with a high antigenic load resulting from exposure to pathogens, pathobionts, and commensal bacteria leading to chronic mucosal immune activation (6). A combination of pro-inflammatory dysbiotic microbiota and exaggerated mucosal immune activation due to intestinal leak in PD appears to be the underlying mechanism of intestinal inflammation in PD. Examination of colonic biopsy tissue from PD patients demonstrate high levels of pro-inflammatory cytokines (TNFα, IL-1β, IFNγ, IL-5) (7). Production of these cytokines is important because co-culture of autologous Th17 cells with dopaminergic neurons showed that Th17 cell production of IL-17A damage DA neurons resulting in cell death (8). GI tract inflammation is a feature associated with PD, even during early stages of the disease, and this mucosal immune activation/inflammation may trigger and/or sustain neuroinflammation is required for α-synuclein aggregation and loss of dopaminergic neurons in PD. Taken together, these findings provide compelling evidence to support the view that the dysbiotic intestinal microbiota is a trigger and/or enabler for the sustained neuroinflammation that can initiate and/or promote PD pathogenesis.

Among the lifestyle factors most strongly implicated in PD pathogenesis are diet and physical activity. Consumption of a Western diet is a risk factor for PD whereas diets high in fiber are associated with reduced risk. While diet has many effects on the body (e.g., omega-3-fatty acids, polyphenols), diet robustly impacts the intestinal microbiota. Consumption of a Western diet promotes pro-inflammatory intestinal microbiota dysbiosis, characterized by a high relative abundance of LPS-containing bacteria and low relative abundance of SCFA-producing bacteria (4), which can promote neuroinflammation. Additionally, there is a growing body of evidence that physical activity reduces the risk of developing PD (9) and ameliorates Parkinsonism symptoms (10–15), and quality of life (16). Imaging studies demonstrate that exercise is associated with greater release of DA in the ventral and dorsal striatum, increases neurotrophin levels, improves vascularization, facilitates synaptogenesis, reduces inflammation, and reduces disordered protein deposition (15). It is not clear how physical activity mediates these beneficial effects on PD patients, but it may include changes in the microbiota. For example, physical activity is associated with a reduction in pro-inflammatory LPS-containing bacteria and an increase in SCFA-producing bacteria. It is possible that both diet and physical activity via changes in the microbiota modulate neuroinflammation and PD pathogenesis via changes in immune function.

How does dysbiotic microbiota trigger/promote neuroinflammation? The microbiota has many features that can influence inflammation, but compelling evidence indicates that low SCFA and high LPS [via binding to toll like 4 receptor (TLR) leading to activation of the NLRP3 inflammasome] are important. SCFA are thought to be anti-inflammatory with an inverse relationship observed between SCFA levels and pro-inflammatory cytokines such as IL-6, IL-12, and TNF-α (17). SCFA can cross the blood brain barrier and microglia (resident immune cells in the brain) are influenced by SCFA (18). SCFA (especially butyrate) are essential for health of intestinal colonic epithelial cells (19) and low SCFA are associated with disrupted intestinal barrier with a concurrent increase in LPS in the systemic circulation (20). There is also a substantial amount of data demonstrating the importance of the LPS-induced NLRP3 inflammasome activation in PD (21). Activation of the NLRP3 inflammasome following exposure to microbial (e.g., LPS or damage-associated stimuli) induces robust inflammation (e.g., production of IL-1β). NLRP3 levels increase in response to factors such as consumption of a Western diet and it could be that this primes the immune system to respond to increased LPS (as is the case of microbiota dysbiosis) (22). These mechanisms (and others) might be the underlying mechanism for impact of the diet and physical activity on PD pathogenesis and disease course.

This special issue will present evidence demonstrating the critical role of the intestinal microbiota and the immune system in PD pathogenesis, as well as how diet and physical exercise might impact PD disease course (via a mechanism including an altered microbiota). This special issue highlights the potential importance of the bi-directional relationship of the brain and intestinal microbiota and microbiota/immune system in PD underscoring the need to better understand the microbiota-gut-brain axis in PD in order to identify potential therapeutic target(s) to design scientifically-based, gut/microbiota-directed disease modifying therapeutics to prevent and/or treat PD and positively impact PD disease course.

## Author Contributions

GA conceived the idea of this topic and managed most of the reviews. Consequently, he developed a first draft of this contribution, reporting the highlights of the topic, and gave specific aspects about physical activity. AK supervised this work and gave a significant contribution about the role of GA. SA supervised the contribution and offered specific aspects about immune system. All authors discussed the results and contributed to the final manuscript.

## Conflict of Interest

The authors declare that the research was conducted in the absence of any commercial or financial relationships that could be construed as a potential conflict of interest.
